# Prevalence of pre-diabetes and type 2 diabetes mellitus among Sami and non-Sami men and women in Northern Norway – The SAMINOR 2 Clinical Survey

**DOI:** 10.1080/22423982.2018.1463786

**Published:** 2018-04-26

**Authors:** Ali Naseribafrouei, Bent-Martin Eliassen, Marita Melhus, Johan Svartberg, Ann Ragnhild Broderstad

**Affiliations:** a Centre for Sami Health Research, Department of Community Medicine, Faculty of Health Sciences, UiT The Arctic University of Norway, Tromsø, Norway; b Division of Internal Medicine, University Hospital of North Norway, Tromsø, Norway; c Tromsø Endocrine Research Group, Department of Clinical Medicine, UiT The Arctic University of Norway, Tromsø, Norway; d Department of Medicine, University Hospital of North Norway, Harstad, Norway

**Keywords:** HbA1c, Norwegian, indigenous, native, aboriginal, ethnicity, ethnic minority, abdominal obesity, waist-to-height ratio

## Abstract

The aim of this study was to determine and compare the prevalence of pre-diabetes and type 2 diabetes mellitus (T2DM) among Sami and non-Sami men and women of rural districts in Northern Norway. The SAMINOR 2 Clinical Survey is a cross-sectional population-based study performed in 2012–2014 in 10 municipalities of Northern Norway. A total of 12,455 Sami and non-Sami inhabitants aged 40–79 years were invited to participate and 5878 were included in the analyses. Participants with self-reported T2DM and/or a glycated haemoglobin (HbA1c) result ≥6.5% were categorised as having T2DM. Those with 5.7%≤HbA1c<6.5% were categorised as pre-diabetics. In men, the total age-standardised prevalence of pre-diabetes (37.9% vs 31.4%) and T2DM (10.8% vs 9.5%) were higher in Sami compared with non-Sami; the ethnic difference was statistically significant for both pre-diabetes (OR 1.42, *p* < 0.001) and T2DM (OR 1.31, *p* = 0.042). In women, pre-diabetes (36.4% vs 33.5%) and T2DM (8.6% vs 7.0%) were also more common in Sami than non-Sami; the differences in both pre-diabetes (OR 1.20, *p* = 0.025) and T2DM (OR 1.38, *p* = 0.021) were also statistically significant. The observed ethnic difference in the waist-to-height ratio (WHtR) was a plausible explanation for the ethnic difference in the prevalence of pre-diabetes and T2DM.

## Introduction

The prevalence of type 2 diabetes mellitus (T2DM) is increasing globally. In 2014 it was estimated that 422 million people worldwide were affected by the disease, and the prevalence of diabetes mellitus (DM) among adults over 18 years of age reached to 8.5% [1]. In 2011, the direct costs of DM treatment in Norway amounted to €408 million; indirect costs amounted to €108 million [2]. There has been no nation-wide survey from Norway on the prevalence of diabetes, but in 2013, it was reported that 2.7% of the country’s population was being treated with glucose-lowering medications [3], and the annual number of new users of glucose-lowering medications in Norway levelled off in recent years [4]. However, there are many individuals who remain undiagnosed of T2DM, or who received a diagnosis but manage their T2DM solely by changes in diet and/or physical activity [5].

The Sami are an indigenous people whose traditional settlement area (Sápmi) covers the northern parts of Norway, Sweden and Finland, and the Kola Peninsula of Russia [6]. However, many Sami are today settled outside Sápmi, especially in larger cities [7]. No valid or updated demographic record of the Sami exists. However, rough estimates of the total number of Sami tend to vary between 50,000 and 100,000, of whom 40,000–50,000 are settled in Norway [8]. The Sami harbour a rich variety of cultures, traditions and languages, but for many decades they were subjected to discrimination and assimilation policies; consequently, many Sami abandoned their native culture and language [9].

The Population-based Study on Health and Living Conditions in Regions with Sami and Norwegian Populations (the SAMINOR Study) aims to investigate the health and living conditions of the Sami and non-Sami people in northern parts of Norway. While the prevalence of lifestyle-related diseases and metabolic syndrome is generally higher among indigenous people as compared to general populations [10,11], studies based on data from the SAMINOR 1 Survey (2003–2004) and the SAMINOR 2 Clinical Survey (2012–2014, hereinafter referred to as SAMINOR 2), found overall high, yet rather similar prevalence of DM in the Sami and non-Sami populations [12–14]. In these studies, DM was recognised by self-report and/or non-fasting plasma glucose measurements. Nevertheless, further studies on the prevalence of DM in these populations, using a more reliable and valid disease indicator, are warranted.

Therefore, the aim of this study was to use HbA1c measurements together with self-reported T2DM collected in SAMINOR 2 to determine and compare the prevalence of pre-diabetes and T2DM among Sami and non-Sami men and women of rural districts in Northern Norway.

## Methods

**Figure 1.**
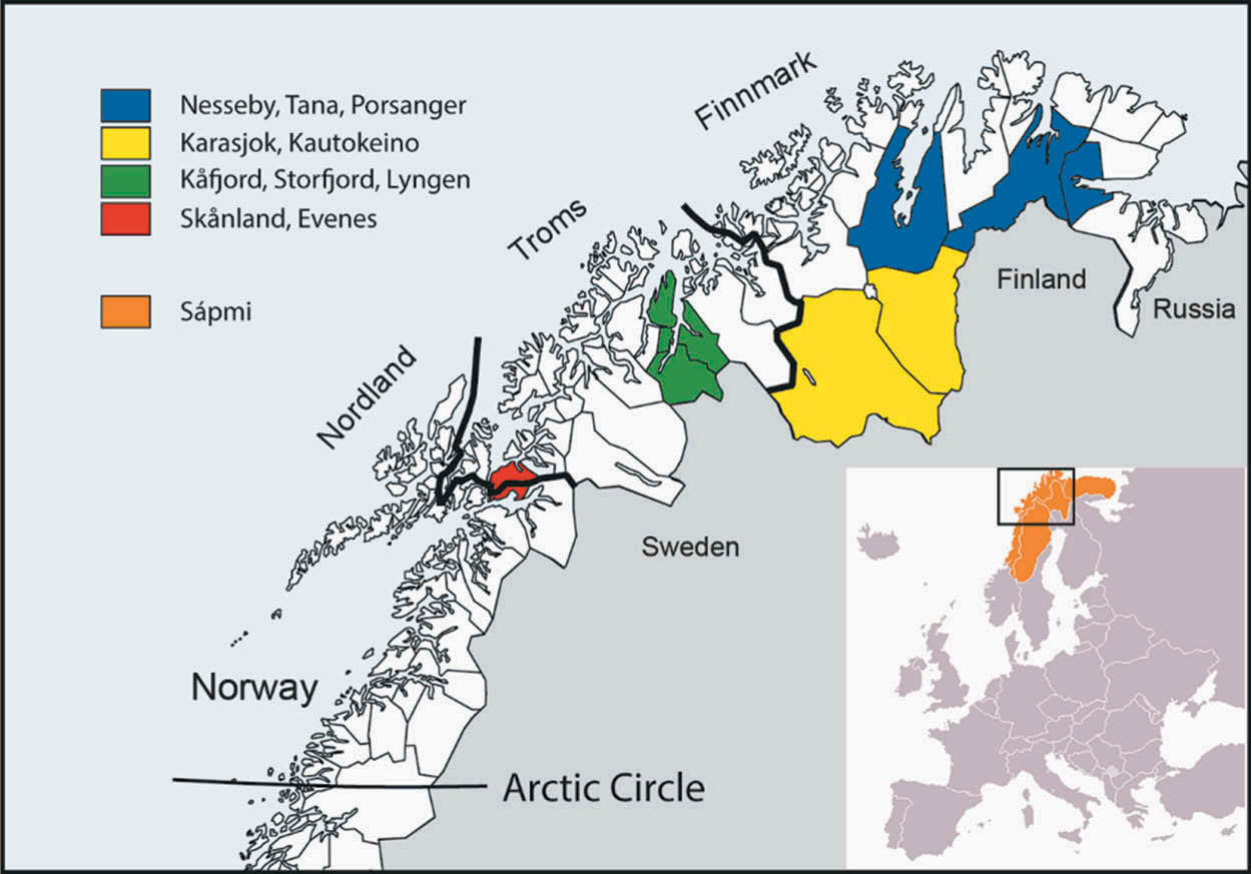
Map of Northern Norway, Sápmi and the included municipalities in the SAMINOR 2.

The present analyses are based on cross-sectional data from SAMINOR 2 which was conducted by the Centre for Sami Health Research at UiT The Arctic University of Norway in 2012–2014. The survey included inhabitants from ten of the municipalities of Finnmark, Troms, and Nordland counties: Kautokeino, Karasjok, Porsanger, Tana, Nesseby, Lyngen, Storfjord, Kåfjord, Skånland and Evenes (Figure 1). All inhabitants in the selected region (i.e. registered in the National Registry of Norway as resident in one of the mentioned municipalities) aged 40–79 years (*n* = 12,455) were invited to participate, regardless of ethnic background. The survey included a self-administered questionnaire and a clinical examination, including collection of a blood sample. Of the 12,455 inhabitants, 6004 (48.2%) attended. Of these 6004, 21 were excluded due to uncompleted questionnaires, 22 were excluded due to missing glycated haemoglobin (HbA1c) results, 72 participants were excluded due to missing ethnicity variable, and 11 with type 1 DM were excluded. Hence, 5878 individuals (47.2%) were included in the analyses. The selected municipalities were divided into three different regions: “Region 1” comprised of areas in the inland of Finnmark County, including Kautokeino and Karasjok municipalities. “Region 2” consisted of both inland and coastal areas in Finnmark County, including Porsanger, Tana and Nesseby municipalities. “Region 3” was made up of the remaining municipalities, all located in Troms and Nordland counties (Lyngen, Storfjord, Kåfjord, Skånland and Evenes) (Figure 1).

The SAMINOR Study was approved by the Norwegian Data Inspectorate and by the Regional Committees for Medical and Health of Research Ethics North (REC North). The committee also approved the present study. All participants gave written informed consent for medical research.

### Data collection

Invitations were mailed several weeks before the clinical examinations started in each municipality. The invitation contained relevant information about the survey, including the time and place of the clinical examination, and the study questionnaire. Participants were to hand in their completed questionnaires at the time of the clinical examination, which was performed at one of ten research stations established in nine municipalities (two research stations were set up in Kåfjord, while participants living in Evenes visited the research station in neighbouring Skånland). All clinical examinations were performed within 2–7 weeks in each municipality.

During the clinical examination, trained personnel measured participants’ height (to the nearest 0.1 cm) and weight (to the nearest 100 g) using an electronic height and weight scale (DS-103, Dongsahn Jenix, Seoul, Korea) with participants wearing light clothing and no shoes. These measures were then used to calculate body mass index (BMI, kg/m^2^). Waist circumference (WC) was measured at the umbilicus to the nearest cm with the participant standing and breathing normally. Waist-to-height ratio (WHtR) was calculated by dividing waist circumference by height. Whole blood samples collected by venipuncture were used for HbA1c testing using DCA Vantage™ (Siemens Medical Solutions Diagnostics, Tarrytown, NY, USA).

Questionnaires differed by age group: participants aged 40–69 years received an 8-page questionnaire that covered a broad range of questions on lifestyle, diet, risk factors and diseases. In contrast, participants aged 70–79 years received a 4-page questionnaire with fewer questions and larger fonts. The present study only included questions that were identical in the two questionnaires. Both questionnaires were originally prepared in Norwegian and then translated into the Northern Sami language. The questionnaires, also a translated English version of the 40–69 year questionnaire, may be reviewed at www.saminor.no. In Kautokeino, Karasjok, Nesseby, and Tana, invitees received both the Sami and Norwegian versions of the questionnaire. In Kåfjord, Storfjord, Porsanger and Lyngen, the Sami version was available on request. Invitees in Skånland and Evenes received the Norwegian questionnaire only. Among all of our participants, less than 5% chose to use the Sami version of the questionnaire.

Information on ethnicity was recorded based on participants’ answers to the following questions: “What language(s) do/did you, your parents and your grandparents use at home?”, “What is your, your father’s and your mother’s ethnic background?”, and “What do you consider yourself to be?” The response options were: “Norwegian”, “Sami”, “Kven” (another national ethnic minority group) [15] and “Others”. Participants were to apply the information for each of the mentioned relatives separately, and multiple languages/ethnicities were allowed. Participants were defined as Sami if they responded that they either considered themselves to be Sami or reported to have a Sami ethnic background, and if in addition at least one of their grandparents, parents, or they themselves spoke a Sami language at home. All participants who did not meet this criterion were defined as non-Sami.

Information on DM was taken from both questionnaires and HbA1c results. First, self-reported type 2 diabetics were ascertained. In the questionnaire this information came from the question: “Have you ever been diagnosed with diabetes (elevated blood sugar levels)?” The available answers were “yes” or “no”. Missing values were classified as “no”. If the participant answered “yes”, they were asked about the type (type 1 DM, T2DM, or gestational diabetes). In addition to participants who reported T2DM, those who reported DM without specifying the type (56 participants) were also categorised as having T2DM. Moreover, those who reported having type 1 DM (T1DM), but reported taking glucose-lowering medication for its treatment (26 participants) or never using insulin (6 participants), were recategorised as having T2DM.

In addition to self-report, those with HbA1c ≥6.5% were also categorised as having T2DM. As virtually all individuals with T1DM are aware of their disease and are under treatment, all those who had high HbA1c (≥6.5%) without reporting diabetes in the questionnaires were regarded as having T2DM. Those who had 5.7%≤HbA1c<6.5% were categorised as pre-diabetics. The pre-diabetes category was defined based on HbA1c only.

Participants gave information on their level of physical activity on a scale of one (very low) to ten (very high). The participants were informed that household chores and professional activities as well as regular exercise and other physical activity, such as walking/hiking, should be taken into account when answering. This scale was validated in middle-aged women in Tromsø, Norway [16]. Educational attainment was reported in the questionnaire in years, and all completed school years were counted.

### Statistical analysis

The data management and statistical analysis were done using STATA version 14.1 (StataCorp, College Station, TX, USA). Differences in mean age, education, physical activity score, height, weight, WHtR, BMI, and WC by sex and ethnic groups were assessed using two-sample *t*-tests. The prevalence of self-reported T2DM and categorised HbA1c was compared between groups using χ^2^ tests (Table 1). Prevalence of pre-diabetes and T2DM is presented as percentages with 95% confidence intervals (CIs) by sex, age and for Sami versus non-Sami participants (Table 2). Due to large samples, CIs were calculated based on normal approximation. The direct method was used to age-standardise the prevalence of pre-diabetes and T2DM. To obtain estimates that better reflect the true prevalences in the selected municipalities and age groups, invitees in SAMINOR 2 were chosen as the standard population (age groups: 40–59, 60–69 and 70–79 years). Multinomial logistic regression analysis was used to calculate the odds ratio (OR) of pre-diabetes and T2DM for Sami compared to non-Sami, stratified by sex. For each sex, five models were run with dysglycaemia as dependent variable and ethnicity (Sami vs. non-Sami) as independent variable: In addition to ethnicity, the first model adjusted for age as continuous variable. In addition to age, the next four models also adjusted for each of the variables physical activity, education, BMI and WHtR, one at a time (Table 3). All these variables were treated as continuous. Comparison between men and women was also performed using multinomial logistic regression adjusted for age. All tests were two-sided with a 5% significance level.

**Table 1.** Crude characteristics of participants in the SAMINOR 2 Clinical Survey (2012–2014), *n* = 5878^a^.

	Total	Sami	Non-Sami	
	*n* = 2688	*n* = 1114	*n* = 1574	*p*-Value^b^
Men				
Age (years)	60.1	59.9	60.3	0.25
Education (years)	11.7	11.4	11.8	0.01
Physical activity (self-rated score)	5.2	5.1	5.2	0.24
Height (cm)	173.1	170.1	175.2	<0.01
WC (cm)	99.6	98.7	100.3	<0.01
WHtR	0.576	0.580	0.572	<0.01
Weight (kg)	84.7	82.0	86.6	<0.01
BMI (kg/m^2^)	28.2	28.3	28.2	0.36
HbA1c<5.7%, *n* (%)	1456 (54.2)	556 (49.9)	900 (57.2)	0.01
5.7%≤HbA1c<6.5%, *n* (%)	1001 (37.2)	456 (40.9)	545 (34.6)	
6.5%≤HbA1c, *n* (%)	231 (8.6)	102 (9.2)	129 (8.2)	
Self-reported T2DM, *n* (%)	254 (9.4)	107 (9.6)	147 (9.3)	0.79
Women	*n* = 3190	*n* = 1282	*n* = 1908	
Age (years)	58.9	58.5	59.1	0.16
Education (years)	12.3	12.5	12.3	0.13
Physical activity (self-rated score)	5.4	5.2	5.6	<0.01
Height (cm)	160.0	156.8	162.2	<0.01
WC (cm)	93.2	93.6	92.9	0.13
WHtR	0.583	0.597	0.573	<0.01
Weight (kg)	71.6	70.0	72.7	<0.01
BMI (kg/m^2^)	28.0	28.5	27.6	<0.01
HbA1c<5.7%, *n* (%)	1776 (55.7)	685 (53.4)	1091 (57.2)	0.11
5.7%≤HbA1c<6.5%, *n* (%)	1232 (38.6)	521 (40.6)	711 (37.3)	
6.5%≤HbA1c, *n* (%)	182 (5.7)	76 (6.0)	106 (5.5)	
Self-reported T2DM, *n* (%)	211 (6.6)	88 (6.9)	123 (6.4)	0.57

Numbers are mean unless stated otherwise. ^a^The number of participants for each variable may differ as some of the measures have missing values. The highest number of missing was for physical activity (*n* = 278). ^b^*p*-values are from two independent samples *t*-test or Pearson chi-square test. WC: waist circumference; WHtR: waist-to-height ratio; BMI: body mass index; HbA1c: glycated haemoglobin; T2DM: type 2 diabetes mellitus.

**Table 2.** Prevalence of pre-diabetes and type 2 diabetes mellitus (T2DM) by sex, age and for Sami versus non-Sami participants. Pre-diabetes is based on 5.7%≤HbA1c<6.5% and T2DM is based on self-report and/or HbA1c≥6.5%.

	Men									
	Sami (*n* = 1114)					Non-Sami (*n* = 1574)				
Age (years)	*n*	Pre-D	% (95% CI)	T2DM	% (95% CI)	*n*	Pre-D	% (95% CI)	T2DM	% (95% CI)
40–59 years	511	168	32.9 (28.8–37.1)	36	7.0 (5.0–9.6)	686	185	27.0 (23.7–30.4)	31	4.5 (3.1–6.3)
60–69 years	388	160	41.2 (36.3–46.3)	61	15.7 (12.2–19.7)	562	195	34.7 (20.8–38.8)	81	14.4 (11.6–17.6)
70–79 years	215	105	48.8 (42.0–55.7)	32	14.9 (10.4–20.3)	326	132	40.5 (35.1–46.0)	57	17.5 (13.5–22.0)
Total crude	1114	433	38.9 (36.0–41.8)	129	11.6 (9.8–13.6)	1574	512	32.5 (30.2–34.9)	169	10.7 (9.2–12.4)
Total age-standardised* (95%CI)			37.9 (35.0–40.8)		10.8 (9.1–12.6)			31.4 (29.1–33.7)		9.5 (8.1–10.9)
	Women									
	Sami(*n* = 1282)					Non-Sami (*n* = 1909)				
Age (years)	*n*	Pre-D	% (95% CI)	T2DM	% (95% CI)	*n*	Pre-D	% (95% CI)	T2DM	% (95% CI)
40–59 years	670	181	27.0 (23.7–30.5)	23	3.4 (2.2–5.1)	933	220	23.5 (20.9–26.4)	31	3.3 (2.3–4.7)
60–69 years	403	182	45.2 (40.2–50.2)	55	13.6 (10.4–17.4)	613	272	44.4 (40.4–48.4)	51	8.3 (6.2–10.8)
70–79 years	209	110	52.6 (45.6–59.6)	35	16.7 (11.9–22.5)	362	173	47.8 (42.5–53.1)	62	17.1 (13.4–21.4)
Total crude	1282	473	36.9 (34.2–39.6)	113	8.8 (7.3–10.5)	1908	665	34.8 (32.7–37.0)	144	7.5 (6.4–8.8)
Total age-standardised^a^ (95%CI)			36.4 (33.9–39.0)		8.6 (7.1–10.0)			33.5 (31.5–35.6)		7.0 (5.9–8.1)

The SAMINOR 2 Clinical Survey (2012–2014), *n* = 5878. ^a^The direct method using the invited sample in the SAMINOR 2 Clinical Survey as the reference population. Pre-D: pre-diabetes; T2DM: type 2 diabetes mellitus; HbA1c: glycated haemoglobin; CI: Confidence interval.

**Table 3.** Odds ratios for pre-diabetes and type 2 diabetes mellitus (T2DM) for Sami compared to non-Sami stratified by sex. The SAMINOR 2 Clinical Survey (2012–2014), *n* = 5878.

	Pre-diabetes			Type 2 diabetes mellitus		
	OR Sami vs. non-Sami	95% CI	*p*-Value^a^	OR Sami vs. non-Sami	95% CI	*p*-Value^a^
Men						
Adjusted for^b^:						
Age	1.42	1.20–1.68	<0.001	1.31	1.01–1.70	0.042
Age + education	1.39	1.16–1.64	<0.001	1.23	0.94–1.61	0.123
Age + physical activity	1.38	1.17–1.65	<0.001	1.26	0.96–1.65	0.093
Age + BMI	1.41	1.18–1.67	<0.001	1.31	1.00–1.71	0.050
Age + WHtR	1.36	1.14–1.62	<0.001	1.19	0.91–1.56	0.197
Women						
Adjusted for^b^:	OR Sami vs. non-Sami	95% CI	*p*-Value^a^	OR Sami vs. non-Sami	95% CI	*p*-Value^a^
Age	1.20	1.02–1.41	0.025	1.38	1.05–1.82	0.021
Age + education	1.21	1.02–1.43	0.023	1.41	1.06–1.88	0.017
Age + physical activity	1.19	1.01–1.40	0.040	1.29	0.96–1.73	0.094
Age + BMI	1.12	0.95–1.32	0.166	1.22	0.92–1.63	0.166
Age + WHtR	1.05	0.89–1.23	0.589	1.00	0.74–1.34	1.00

^a^*p*-values present the statistical significance of the corresponding ORs for pre-diabetes or T2DM vs. normoglycaemics.

^bNumber of individuals in each regression analysis may vary due to some missing values in each adjusted variable.^

OR: odds ratio; CI: confidence interval; BMI: body mass index; WHtR: waist-to-height ratio.

## Results

Some characteristics of the participants are shown in Table 1. Mean WHtR was higher in Sami men compared to their non-Sami counterparts. In women, both mean BMI and WHtR was significantly higher among Sami compared to non-Sami. On average, Sami women reported significantly lower physical activity than did their non-Sami counterparts (Table 1).

The overall age-standardised prevalence of self-reported T2DM was 7.4% (95% CI: 6.8–8.0) (results not shown). While more men than women reported T2DM, there was observed no ethnic difference in the prevalence of self-reported T2DM (Table 1). In total, 2083 (35.4%) individuals were ascertained as pre-diabetics (5.7%≤HbA1 < 6.5%) and 565 (9.4%) as type 2 diabetics (self-reported T2DM and/or 6.5%≤HbA1c). Of those who were categorised as having T2DM, 465 (82.3% of all cases) reported T2DM themselves (results not shown). The total age-standardised prevalence of pre-diabetes and T2DM were, respectively, 34.1% (95% CI: 33.1–35.1) and 8.7% (95% CI: 8.0–9.4) (results not shown).

In Sami men, the total age-standardised prevalences of pre-diabetes and T2DM were 37.9% and 10.8%, respectively. Corresponding numbers for non-Sami men were 31.4% and 9.5% (Table 2). The 95% confidence intervals of T2DM prevalence overlapped, but as this does not rule out statistical significance, multinomial logistic regression was performed. When adjusting for age as a continuous variable in a multinomial logistic regression, the ethnic difference was statistically significant for both pre-diabetes (OR 1.42, *p* < 0.001) and T2DM (OR 1.31, *p* = 0.042) (Table 3). In women, the age-standardised prevalences of pre-diabetes were 36.4% in Sami vs 33.5% in non-Sami and of T2DM 8.6% in Sami vs 7.0% in non-Sami (Table 2). The ethnic differences in both pre-diabetes (OR 1.20, *p* = 0.025) and T2DM (OR 1.38, *p* = 0.021) were also herein statistically significant (Table 3).

Adjustment for WHtR had the largest impact on the OR for pre-diabetes and T2DM for Sami compared to non-Sami, especially in women (Table 3); after adjusting for WHtR, the OR for pre-diabetes in Sami versus non-Sami women was 1.05 (*p* = 0.589) and for T2DM 1.00 (*p* = 1.00).

In men, the observed prevalence of pre-diabetes and T2DM was higher in Sami in all geographical regions; statistically significant ethnic difference was, however, only found for pre-diabetes in region 2 and for T2DM in region 3 (results not shown).In women, the observed prevalence of pre-diabetes and T2DM was higher in Sami in all geographical regions but region 2, wherein fewer Sami had dysglycaemia. Statistically significant ethnic difference was, however, only observed for pre-diabetes in region 1 and for T2DM in regions 1 and 3 (results not shown).

## Discussion

The overall age-standardised prevalence of pre-diabetes and T2DM in the 10 municipalities were, respectively, 34.1% and 8.7%. In spite of overlapping confidence intervals of age-standardised prevalence of pre-diabetes (in women) and T2DM (in both sexes) of Sami versus non-Sami participants, the age-adjusted ORs of pre-diabetes and T2DM for Sami versus non-Sami were statistically significant in both sexes. Furthermore, the prevalence of T2DM was statistically significantly higher in men. Ethnic differences in WHtR seems to be a plausible explanation for ethnic difference in pre-diabetes and T2DM, especially in women as it explained the entire ethnic difference in pre-diabetes and T2DM.

Self-report of T2DM in combination with HbA1c results were used to categorise participants as having T2DM. HbA1c results reflect average plasma glucose concentration during the preceding 2–3 months [17]. The firm association between HbA1c results and late complications of DM was first documented in a Norwegian study [18]. Due to its high pre-analytical stability, high reproducibility, less day-to-day perturbations during periods of stress and illness, and convenience (no need for prior fasting or glucose overload), HbA1c is being increasingly utilised in medical settings for both diagnosis and follow-up of patients with DM [5]. In 2009, the International Expert Committee recommended the use of HbA1c to diagnose DM. However, they stressed that there was a continuum of risk for DM across HbA1c results [19], admitting that, although the risk of retinopathy escalates drastically at HbA1c ≥6.5%, the risk of developing DM and its other complications may clearly begin well under this cut-off [19].

In our study, more than one third of participants were diagnosed as having pre-diabetes. The American Diabetes Association recommend HbA1c≥5.7 for pre-diabetes [5]. The sensitivity of this cut-off is also quite low [20,21]. The American Diabetes Association recommends that individuals with HbA1c levels of 5.7–6.4% be informed of their increased risk for DM and cardiovascular diseases and counselled about effective preventive strategies such as weight reduction and increased physical activity [5]. It should be kept in mind that the risk of developing DM follows a continuum of risk rather than a certain cut-off [5]. However, different guidelines recommend that clinicians have two HbA1c results ≥6.5% to establish a diagnosis of DM [5,19,22,23]. In the Tromsø OGTT Study, the sensitivity, specificity, positive and negative predictive values for HbA1c ≥ 6.5% were, respectively, 34.7%, 97.1%, 41.2% and 96.1% using OGTT (oral glucose tolerance test) as gold standard [24]. As both the sensitivity and specificity of the HbA1c test are <100%, a misclassification in the outcome variable (T2DM) can be expected. This misclassification is most likely non-differential with regard to ethnic groups.

In this study, questionnaires were applied to acquire information on T2DM. As the performance of questionnaires may be affected by issues like recall bias, unawareness of the disease, or misinterpretation of the questions, self-reported data may be inadequate to reflect the true prevalence of a disease. In a study performed in Olmsted County, Minnesota, with 2037 participants aged ≥45 years, the sensitivity and positive predictive value of self-reported DM were 66.0% and 94.3%, respectively [25]. However, the CADEUS study in France reported a sensitivity and positive predictive value of self-reported DM of 86.7% and 73.4%, respectively [26]. All the mentioned studies used medical records as reference standard. It should be noted, however, that the phrasing of questions and types of criterion standard affect the sensitivity and positive predictive value of questionnaires [27]. Furthermore, some publications have reported that the Sami people may be more inclined than non-Sami to underreporting diseases due to some cultural differences and/or language barriers (differential misclassification) [28].

The ethnicity (exposure variable) of the participants was ascertained based on the obtained data from the questionnaires. Contrary to reporting non-Sami ethnicity, reporting Sami ethnicity demands a conscious choice. Due to decades of stigmatisations and histories of study misconduct exerted on the Sami people, there are still some Sami people who are hesitant to either participate in such studies or report their ethnicity as Sami. As a result, some Sami people may have been misclassified as non-Sami, while the opposite is extremely unlikely. This leads to a non-differential misclassification in the exposure variable. The joint effect of the mentioned misclassifications in the exposure and outcome variables might have diluted the measure of association in our study [29]. It is possible that the real difference between the Sami and non-Sami with regard to the prevalence of T2DM was higher than what was observed.

In our study, the estimated age-standardised prevalence of self-reported T2DM was 7.4%. Data from the Norwegian Prescription Database show that in 2014, 6.8% of inhabitants aged 40–79 years in the 10 municipalities included in our study were using oral glucose-lowering medications for T2DM [14]. This may serve as a validation of our estimate of known cases of T2DM in the study population.

The observed difference in the prevalence of pre-diabetes and T2DM between Sami and non-Sami of the same sex in the present study is in discordance with results from previous studies [12–14,30]. This might be due to our use of HbA1c as the diagnostic test in contrast to previous studies which were based on self-report and/or non-fasting (random) plasma glucose. This is supported by the fact that our study showed no ethnic difference in the prevalence of self-reported T2DM. However, the observed ethnic discrepancy may also be attributed to various genetic, biological, environmental, and lifestyle-related risk factors. It should also be mentioned that some of the previous publications are based on data from a larger geographic area than our study.

Adjustment for WHtR in the multinomial logistic regression analysis diminished or eliminated the ethnic difference in the prevalence of pre-diabetes and T2DM, and this impact was most striking for T2DM among women (Table 3). It should be mentioned that as Sami people are generally shorter in stature than their non-Sami counterparts (Table 1), it is more appropriate to use WHtR than WC. According to Table 1, both BMI and WHtR in women, and WHtR in men, were higher among Sami individuals. It is believed that adipose tissue in obese people releases higher amounts of non-esterified fatty acids, glycerol, hormones, pro-inflammatory cytokines and other factors which play an important role in the development of dysglycaemia and eventually T2DM [31]. Higher prevalence of obesity (especially abdominal) and its implication in the higher prevalence of T2DM among indigenous peoples have been reported in a number of publications [32–34].

In the present study, there was observed higher prevalence of pre-diabetes and T2DM among Sami compared to their non-Sami counterparts in almost all geographical regions. However, due to small numbers in each region, only very large differences would have been statistically significant.

Traditionally, most of the population in Northern Norway has relied on primary industries, such as small-scale farming and fishing, and for parts of the Sami population: reindeer herding. A combination of these industries were common. Today, fewer people work in primary industries; instead the number of people employed in service industries has grown. Fewer people have physically active jobs, and even farming and reindeer herding are largely reliant on motor-vehicle transport. This transition from a physically-demanding to a more sedentary lifestyle, which has taken place in both Sami and non-Sami populations, may have increased the risk of developing T2DM [35,36].

Whereas publications on the prevalence of T2DM among other Arctic indigenous populations in the age span 40–79 years are rather sparse, there is compelling evidence that indigenous peoples still suffer from poorer health and social outcomes than do benchmark populations in most countries [37]. There are numerous studies reporting that the prevalence of T2DM and some other lifestyle-related chronic diseases in indigenous peoples are generally either higher than benchmark populations or on the rise. For example, the prevalence of T2DM among Greenland Inuit (age≥18 years) in 2005–2010 was reported to be 9%, of which 79% were previously unknown cases [38]. The overall prevalence of T2DM among Canadian Inuit was in 2006 comparable to the general Canadian population (6.8%), while it was around 2% in 2001 [39]. First Nations Aboriginals in Canada were reported to have a much higher prevalence of DM (15.3%) than the Métis (5.8%) and Inuit (4.3%) [40]. However, in 2007–2008 the prevalence of T2DM in Canadian Inuit aged ≥50 years was 12.2% [41]. In 2010, the prevalence of diagnosed DM among American Indians and Alaska Native individuals was over 14% which is higher than any other racial or ethnic group in the USA [42]. Contrary to indigenous people in most other countries, the Sami people in Norway have living conditions and a socioeconomic status that are comparable to those of other Norwegians. This could explain the lack of a huge difference in the prevalence of T2DM between Sami and other Norwegians.

The overall prevalence of T2DM was lower in women compared to men. Although some references do not mention sex as an independent risk factor for T2DM [43], the prevalence of T2DM was reported in several studies to be lower among women [44–46] especially in developed countries [47]. The male excess in the incidence and prevalence of T2DM, which is found in some populations, has been attributed to sex-related differences in insulin sensitivity, consequences of obesity and regional body fat deposition and other contributing factors such as hypertension, smoking and alcohol intake [48,49].

### Strengths and limitations

The strengths of this study include its large total sample size (*n* = 5878) and acceptable participation rate, as well as the use of HbA1c as a diagnostic test, which provided us with valuable estimates of the prevalence of pre-diabetes and T2DM in the inhabitants of the included municipalities. By targeting municipalities with a substantial proportion of Sami inhabitants, we ensured a large proportion of Sami in our sample.

Limited knowledge is at hand regarding non-responders, except that there were more non-responders among men and in the younger age groups. It is also likely that the severely sick had restricted ability to participate in the study and those who were more conscious about their health status had higher tendency to participate (selection bias). Furthermore, it is not certain whether the distribution of ethnic groups in our study reflects the actual ethnic composition of the included municipalities, as there is no ethnic registry in Norway. However, the response was particularly high in some of the municipalities where the Sami are in majority, which may indicate a higher overall response among Sami compared to non-Sami. Only 10 municipalities were included in SAMINOR 2, hence generalisations to the entire Sami or non-Sami populations in Norway is not advised.

Glucose-based tests (fasting plasma glucose and glucose tolerance test) as well as a physical examination to detect signs and symptoms of DM were not performed due to practical issues.

Our definition of ethnicity is not a mutually exclusive one, as individuals might have expressed a sense of belonging to more than one ethnic group. For example if a participant ticked other ethnicity-related options in addition to Sami in the questionnaire, he/she was categorised as Sami. As a consequence of the assimilation policy, many Sami have abandoned their Sami culture and identity, or choose to conceal their background. Therefore, there are participants of Sami descent, who are categorised as non-Sami in our study. Contrary to some other definitions, our definition gave more emphasis to self-identification than linguistic features. As there have been other definitions of Sami ethnicity in the literature, comparison between our results and results from studies with different definitions should be made with caution. The fact that sensitivity analyses performed with different ethnicity definitions produced overall similar results strengthen our findings.

## Conclusion

The overall age-standardised prevalence of pre-diabetes and T2DM were high in the study population. Overall, the prevalence of pre-diabetes and T2DM was higher among Sami compared to their non-Sami counterparts with a higher WHtR in Sami being a plausible explanation. Women in general had lower prevalence of T2DM. Longitudinal studies aiming at assessing the risk of T2DM in Sami and non-Sami, and with a special focus on risk factors such as diet, BMI and WHtR should be undertaken. However, it is at present critical to implement drastic measures in order to reduce the levels of key risk factors and the overall prevalence of T2DM in this population.

## Geolocation information

Europe, Northern Scandinavia, Northern Norway.
